# Nanoparticle Formulation Composition Analysis by Liquid Chromatography on Reversed-Phase Monolithic Silica

**DOI:** 10.1021/acs.analchem.2c04277

**Published:** 2022-12-22

**Authors:** Ekaterina Tsarenko, Ulrich S. Schubert, Ivo Nischang

**Affiliations:** † Laboratory of Organic and Macromolecular Chemistry (IOMC), Friedrich Schiller University Jena, Humboldtstr. 10, 07743 Jena, Germany; ‡ Jena Center for Soft Matter, Friedrich Schiller University Jena, Philosophenweg 7, 07743 Jena, Germany

## Abstract

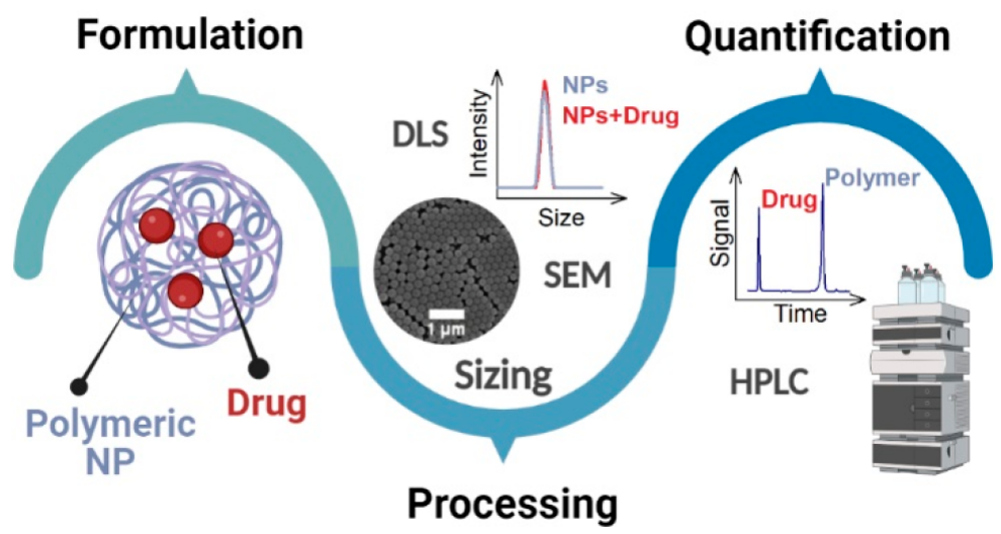

Multifunctional nanoparticle (NP) formulations for medical purposes have already found their way toward envisaged translation. A persistent challenge of those systems is, next to NP size analysis, the compositional analysis of the NPs with the polymer as the matrix component and the encapsulated drug, particularly in a quantitative manner. Herein, we report the formulation of poly(lactic-*co*-glycolic acid) (PLGA) NPs by nanoprecipitation and the analysis of their integrity and size by dynamic light scattering (DLS) and scanning electron microscopy (SEM). Those NPs feature a variety of encapsulated drugs including the well-known ibuprofen (Ibu) as well as dexamethasone (Dex) and dexamethasone acetate (DexAce), with the latter being of potential interest for clinical treatment of SARS-CoV-2 patients. All those dissolved formulation compositions have been subjected to liquid chromatography on reversed-phase silica monolithic columns, allowing to quantitatively assess amounts of small molecule drug and NP constituting PLGA polymer in a single run. The chromatographically resolved hydrophobicity differences of the drugs correlated with their formulation loading and were clearly separated from the PLGA matrix polymer with high resolution. Our study identifies the viability of reversed-phase monolithic silica in the chromatography of both small drug molecules and particularly pharmapolymers in a repeatable and simultaneous fashion, and can provide a valuable strategy for analysis of diverse precursor polymer systems and drug components in multifunctional drug formulations.

The 21st century has brought countless breakthroughs to nanomedicine and in targeted therapeutic drug delivery.^1^ Polymeric nanoparticles (NPs) are, nowadays, under extensive investigation in terms of the targeted delivery of potent drugs or dyes to specific regions in the human body. The choice of polymers allows for tunable hydrophilicity/hydrophobicity, formulation strategies, and surface modification possibilities of the resultant NPs.^2^ Attractive properties such as biocompatibility, biodegradability, and tailored structure by formulation procedures are intensively investigated. A pharmapolymer possessing many of those attractive features, including its use as a scaffold material and for drug delivery purposes, is poly(lactic-*co*-glycolic acid) (PLGA).^3^ It can be considered a “gold” standard pharmapolymer and one of the most frequently used polymers for drug delivery implementations and for NP formulation.^4−6^

While formulation strategies and the encapsulation of a variety of drugs are dominating research on the PLGA pharmapolymer, detailed characterization attempts often have shortcomings in view of the compositional properties of the NPs. Recently, the application of an analytical ultracentrifuge has been reported in the characterization of PLGA NPs with targeting dye moieties and carrying an anti-inflammatory drug. Those studies included NP degradation and drug release dynamics and also reported the determination of encapsulated and free drug.^7^

One of the most important analytical techniques in the pharmaceutical industry comprises high-performance liquid chromatography (HPLC) in the reversed-phase mode. Recent studies mainly focused on either free drug determination or in vivo drug release studies under specific conditions.^8−12^ Typically, an HPLC analysis involves complex sample preparation procedures including filtration or centrifugation while exclusively focusing on the drug, i.e., isolating it from the remainder of the formulation components. We, herein, disclose the potential of reversed-phase monolithic silica that has already demonstrated promising performance in research studies comprising the analysis of small molecules,^13^ and also larger molecules such as the stealth polymers poly(ethylene glycol)^14^ and poly(2-alkyl-2-oxazolines),^15^ while allowing separation selectivity according to the polymer end-group and/or degree of polymerization.

Herein, the applicability of reversed-phase monolithic silica for the quantitative determination of both the NP constituting polymer PLGA and a variety of encapsulated drug components of formulated NPs by fast chromatography is reported. During formulation, the NPs were loaded with the model drug ibuprofen (Ibu), which is a well-known nonsteroidal anti-inflammatory drug that has been on the market for over 50 years,^16^ as well as two anti-inflammatory glucocorticoids–dexamethasone (Dex) and its derivative dexamethasone acetate (DexAce), that lately have been used for potential treatment of hospitalized patients during the COVID-19 pandemic.^17^ A priori, those formulated spherical NPs are thoroughly characterized by dynamic/electrophoretic light scattering (DLS/ELS) and scanning electron microscopy (SEM) regarding their integrity. Simple dissolution of the lyophilized NP formulations including the constituting polymer matrix in a suitable solvent, followed by straightforward chromatography through combination of an isocratic/gradient elution programming, revealed the formulation composition in a straightforward manner.

Scheme 1 shows the chemical structures of the PLGA used for NP formulation and model drugs with varying hydrophobicity (Ibu > DexAce > Dex) (see Section 1.1 of the Supporting Information).

**Scheme 1:**
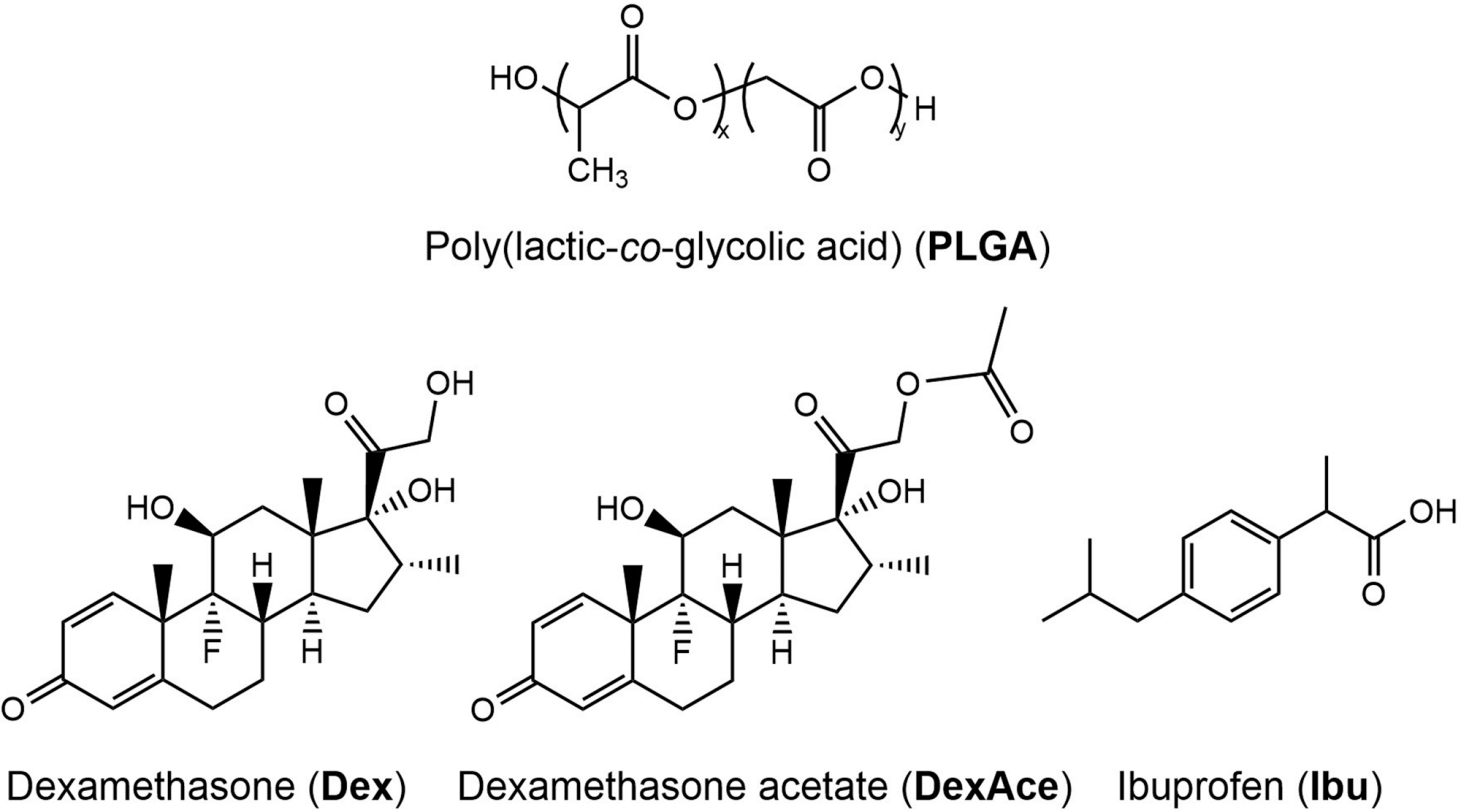
Chemical Structures of Materials Used for NP Formulation

NP formulation (see Section 1.2 of the Supporting Information) and analysis in terms of size and integrity were performed via DLS and SEM (see Sections 1.3 and 1.4. of the Supporting Information). Figure 1A shows that NPs prepared with and without the drug Ibu center at a similar *d*_h,z_ value of ca. 225 nm; i.e., the presence or absence of the drug does not impact the hydrodynamic size estimated by DLS. DLS size distributions for NPs loaded with Dex and DexAce can be found in Figure S1A and B of the Supporting Information. The overall insensitivity of sizes toward drug encapsulation appeared true for all formulations containing the different drugs (Figure 1B) with the DLS size distributions in the intensity mode indicating the absence of large aggregates. Detailed size estimations, *d*_h,z_, their variation calculated from the polydispersity index (PDI), as well as the negative zeta potential (stemming from the acid-terminated PLGA) indicate the integrity of all the formulated NPs in aqueous solution (Table S1 of the Supporting Information). Figure 1C and D demonstrates a similar appearance of the spherically shaped NPs in SEM with and without Ibu, also confirmed by formulations containing the other drugs (see Figure S1C and D of the Supporting Information). Dissolution of lyophilized formulations according to the detailed sample preparation procedure (see Section 1.5 of the Supporting Information) was followed by optimized chromatographic analysis (see Sections 1.6 and 1.7 of the Supporting Information) on a reversed-phase silica monolithic column, featuring micrometer-sized flow through pores of approximately 1.1 μm, confined by a continuous mesoporous C18-derivatized skeleton containing approximately 15 nm sized mesopores. We utilized a combination of an isocratic hold where the drug eluted (mobile phase composition: 60% acetonitrile (ACN)/40% water (v/v)), followed by a steep linear increase toward 100% ACN within 0.25 min. The elution trace exemplified in Figure 2 demonstrates the highly efficient isocratic elution (with plate heights in a range of 9–13 μm, see Table S2 of the Supporting Information) of the drug components (Ibu, DexAce, and Dex) and elution of the disperse PLGA polymer population compressed to a narrow fronting peak, eluting under conditions of 100% ACN in the mobile phase. The chromatographic characteristics such as retention time and peak asymmetry of the analyzed drug components can also be found in Table S2 of the Supporting Information. Those compare well to the literature values previously reported for silica monolithic columns.^18^

**Figure 1:**
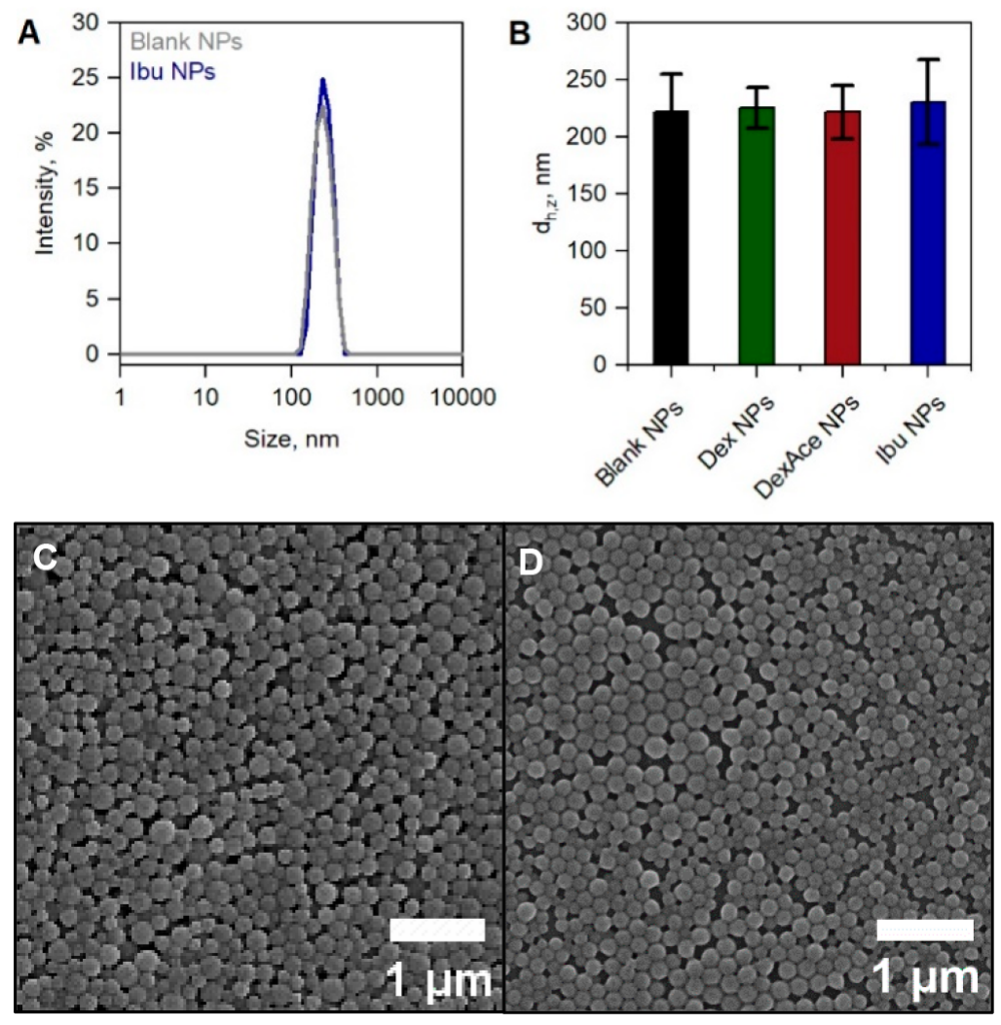
(A) Intensity-based hydrodynamic size distribution of blank PLGA NPs and NPs containing Ibu. (B) Overview of PLGA NP size, *d*_h,z_, and size variations calculated from the PDI values for NPs without and with encapsulated drugs as indicated. (C) SEM image of the PLGA NPs formulated with Ibu and (D) without.

**Figure 2:**
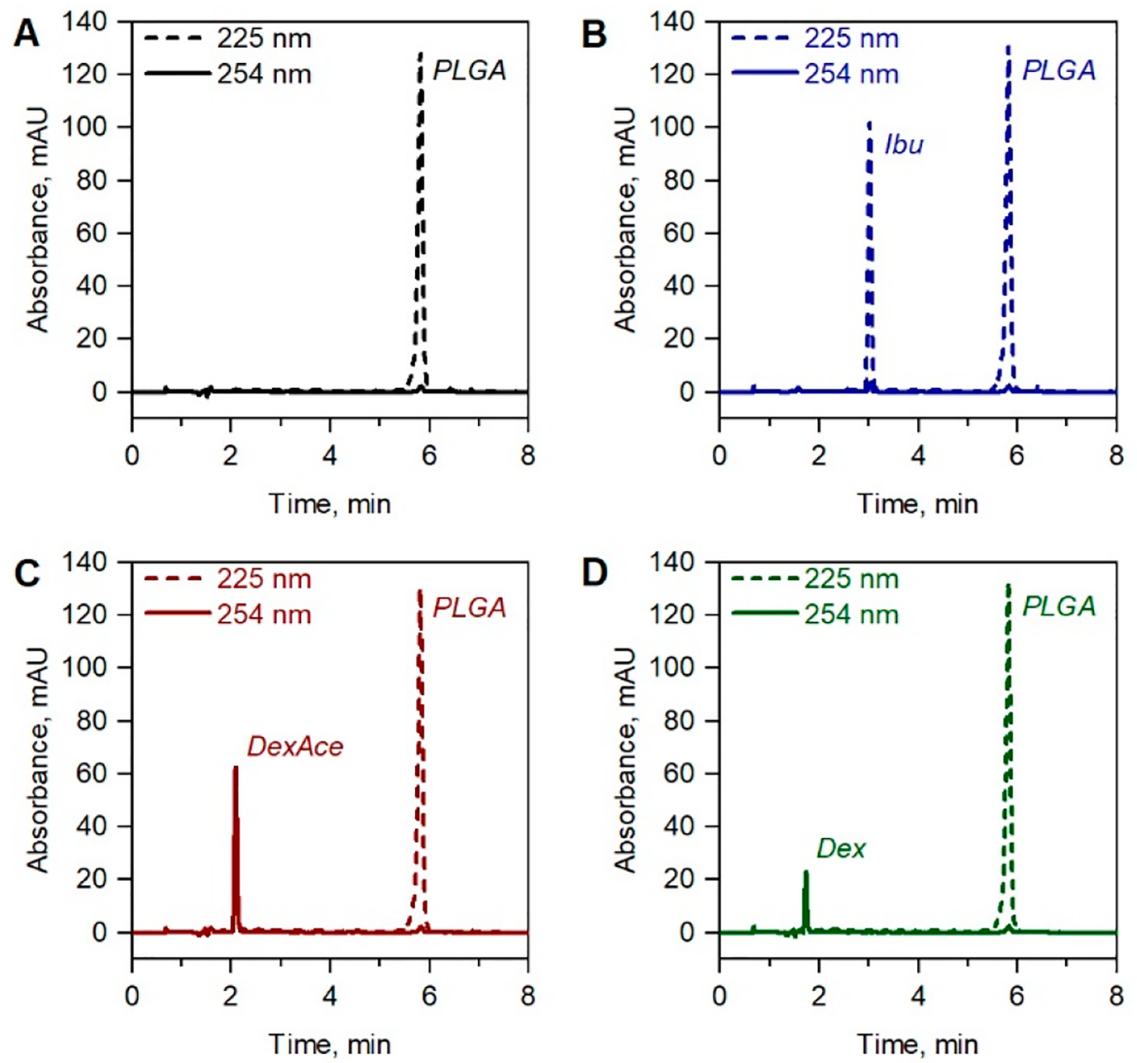
Blank corrected elugrams demonstrating NP composition analysis monitored at a wavelength representative of the drugs and PLGA. (A) Blank PLGA NPs, (B) Ibu-containing NPs, (C) DexAce-containing NPs, and (D) Dex-containing NPs. Elution conditions: flow rate 1 mL min^–1^, isocratic hold for 3 min at 60% (v/v) of ACN in the mobile phase, after which a linear gradient of ACN (from 60% (v/v) to 100%) in 0.25 min was run. UV absorption detection at 225 nm (Ibu and PLGA) and 254 nm (Dex and DexAce).

The elution of the components from the column was monitored via a diode array detector (DAD) operated at two different wavelengths, i.e. 225 and 254 nm. PLGA as well as Ibu have pronounced absorbance intensities at 225 nm (see Figure 2, dashed lines). Before chromatographic analysis, the lyophilized formulations were dissolved in DMSO, followed by addition of ACN and water (see Section 1.5 of the Supporting Information). This results in a tailing and broad elution signal at 225 nm between elution times of 1–2.5 min, interfering with the Dex and DexAce elution signal (see Figure S2 of the Supporting Information). Thus, the elution of Dex and DexAce was monitored simultaneously at 254 nm (Figure 2, solid line) to minimize the interference of the DMSO background elution peak and to enable a clean baseline separation of the solvent and drug elution peak, essential for the envisaged quantitative analysis. Thus, the developed protocol comprising an isocratic hold followed by a steep linear gradient provides the highest efficiency for the drug components (under isocratic conditions) and a selective elution of a fronting PLGA compared to all other elution strategies and gradients that were investigated (see Figure S3 of the Supporting Information).

To demonstrate quantitative analysis of formulation compositions, stock solutions of the drugs (Ibu, Dex, DexAce) and PLGA were prepared according to the established sample preparation procedure (see Section 1.5 of the Supporting Information) and were diluted to a series of different concentrations. Figure 3A represents the elution trace of Ibu and PLGA standards while the Dex and DexAce elution traces can be found in the SI (see Figure S4A and B). The peak areas as a function of concentration (Figure 3B and C) were found to be linear (correlation coefficients were larger 0.999) over the whole concentration range, i.e., 0.1–10.0 mg mL^–1^ for PLGA and 1.0–50.0 μg mL^–1^ for Ibu. A similar quality of data was found for the Dex, and DexAce (see Figure S4C and D of the Supporting Information).

**Figure 3:**
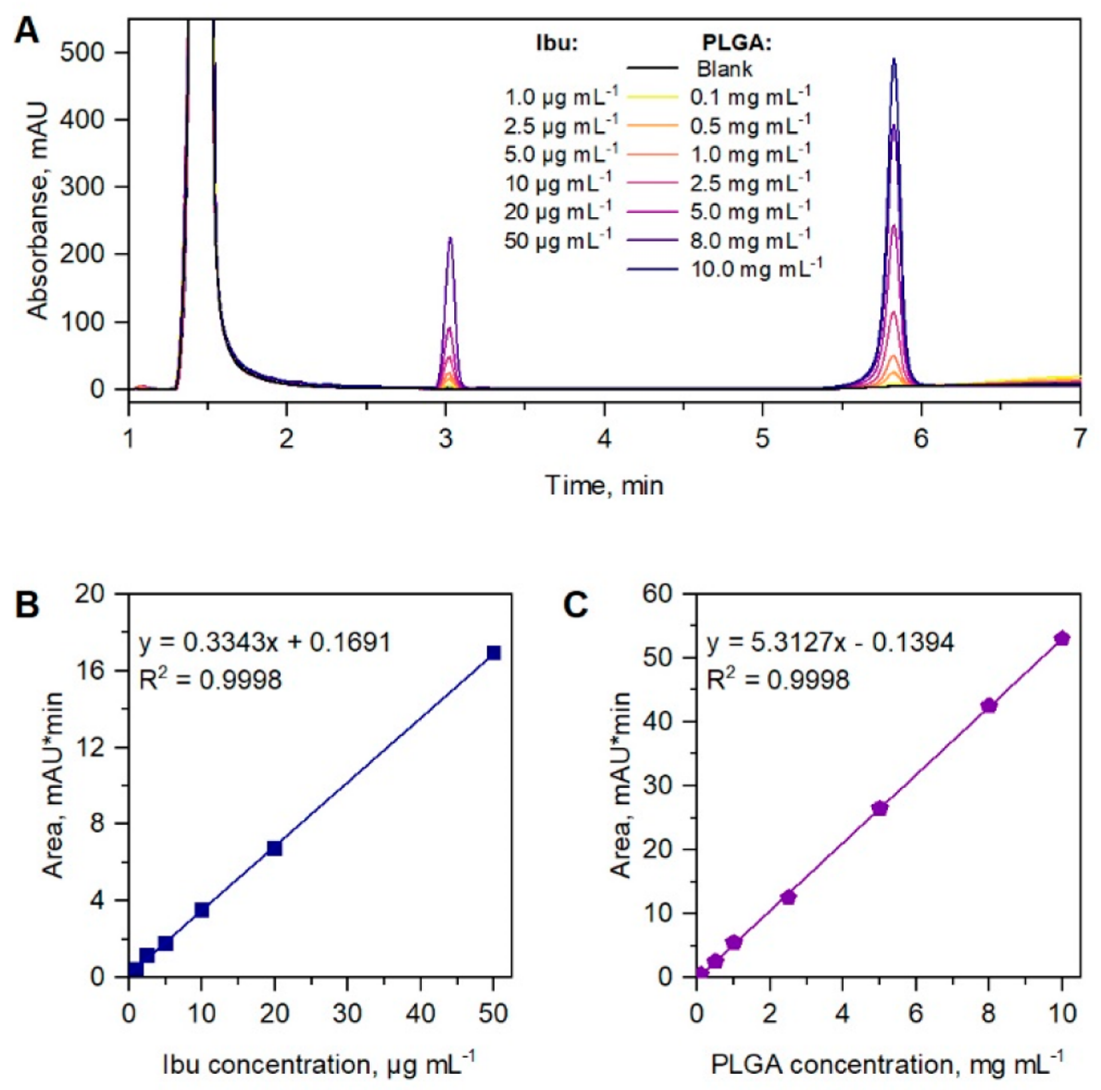
(A) Overlaid elugrams of Ibu (1.0–50 μg mL^–1^) and PLGA (0.1–10.0 mg mL^–1^) samples monitored at a wavelength of 225 nm. Calibration curves for (B) Ibu and (C) PLGA obtained by plotting peak areas as a function of analyte concentrations. Same elution conditions as in Figure 2.

Based on the determined dependencies, concentrations of the polymer as well as the drug components present in the formulations can straightforwardly be calculated as presented in Table S3, with very low standard deviations of repetitive injections. Two basic values for any nanoscale drug delivery system, i.e., encapsulation efficiency (EE) and loading capacity (LC), were calculated according to the equations described in Section 1.8 of the Supporting Information. Assuming all of the drug is encapsulated, both LC and EE values increase in the row: Dex < DexAce < Ibu as seen in Figure 4A. Thus, the encapsulation of the drug in the formulation procedure appears to correlate with its hydrophobicity at a constant composition of the degradable PLGA polymer. At the same time, successively increased hydrophobicity of all retained drugs is apparent via an increased isocratic retention in reversed-phase chromatographic elution comprising 60/40 ACN/water (%, v/v) in the mobile phase (see Figure 4B).

**Figure 4:**
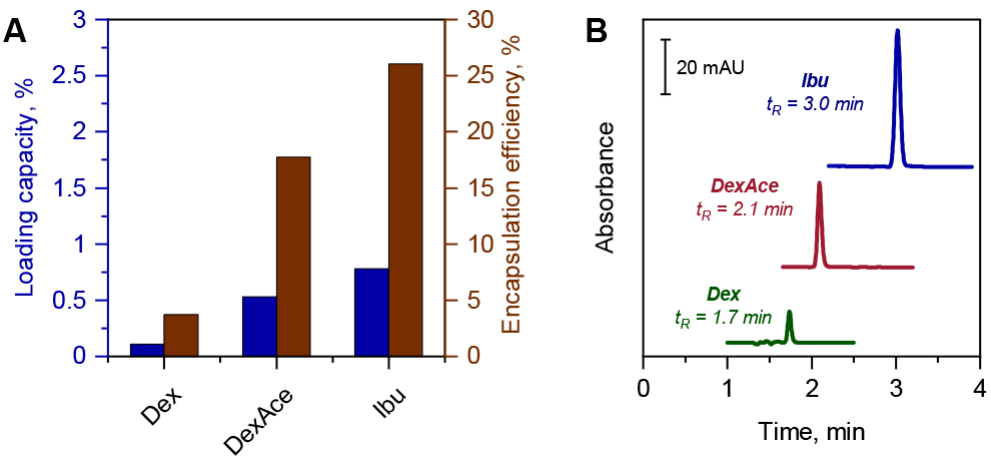
(A) Loading capacities (blue) and encapsulation efficiencies (brown) for drugs with increased hydrophobicity assuming all drug being encapsulated in PLGA NPs. (B) Elugrams of the drugs under same elution conditions as in Figure 2.

While the experimental results in Figure 4A suggest more drug being present in formulations and consequently NPs (according to its hydrophobicity), we performed another series of experiments, in which the lyophilized NPs were resuspended in water followed by centrifugation. Then, the remaining supernatant was lyophilized and analyzed for its drug content by HPLC (see Section 1.5 of the Supporting Information). Interestingly, the amount of “free drug” again scales with its hydrophilicity/hydrophobicity (Figure S5). This shows that “free” and encapsulated drugs can be distinguished. In other words, reports on LC and EE need to be treated carefully, particularly after solution reconstitution of lyophilized samples.

Thereby, the developed analytical concept was demonstrated to enable NP formulation analysis comprising the concentration of drug and potential loading of nanocarriers, together with the NP constituting PLGA. This can be considered a key for optimizing loading capacity and qualifying it in a quantitative manner by taking in account both the drug and PLGA simultaneously.

To gauge repeatability and sensitivity of the reported analytical protocol, another batch of PLGA NPs loaded with Ibu was prepared and the amount of PLGA and Ibu was determined as for the previous batch.

Clearly, the amount of Ibu varied compared to the completely independent previous batch, demonstrating repeatability issues of the NP formulation procedure (Table S4 of the Supporting Information). To show this, the average amount of Ibu and PLGA determined through repetitive injections reveals again a very low standard deviation with a coefficient of variation of 0.20% for Ibu and 0.13% for PLGA. Spiking experiments (see Section 1.5 of the Supporting Information) of both Ibu and PLGA indicate high recoveries (see Tables S4 and S5 of the Supporting Information). Also, filtration of samples prior to analysis appears to only very moderately influence analytical results (Table S6 of the Supporting Information). This demonstrates sensitive analysis of both PLGA and drug components in a quantitative manner. Last but not least, the results highlight the sensitive analysis of NP formulations that can vary from batch to batch and are highly desirable for quality control purposes of NPs in applications.

Summarizing, we have demonstrated the high potential of reversed-phase monolithic silica columns in both qualitative and quantitative analysis of formulated nanoscale drug delivery system components. The column material allowed for the simultaneous separation of both small drug components according to their hydrophobicity in an efficient manner and, simultaneously, a pharmapolymer, straightforwardly and repeatable. The quantitative determination of the drug and the NP constituting polymers in a single chromatographic run, with elution times according to drug hydrophobicity, holds promise for assuring product homogeneity and outcome. The impetus remains to utilize this conceptual analytical approach for applications in fast and robust analysis of NP carrier elements. This is because the quantitative study of structure–property relationships of the NP constituting polymers according to drug hydrophobicity/hydrophilicity are needed to optimize LC and EE of the to-be-designed carriers. This can enable screening of libraries of polymers for NP formulation strategies in direct accordance of the to-be-encapsulated drugs. It is likely that the here described strategy also works for other drug-containing (assembled) polymeric NPs that are molecularly dissolvable and with properly adjusted chromatographic conditions on this type of stationary phase.

Concerning quantitative aspects of drug delivery system analysis, it has been demonstrated that an analytical ultracentrifuge^7^ and here HPLC can deliver quantitative results. While in an analytical ultracentrifuge the NPs are investigated in situ as formulation components in solution, HPLC requires molecular dissolution for sample preparation. A quantitative comparison between an analytical ultracentrifuge and HPLC can decipher the advantages and disadvantages of each of those techniques and can allow for a robustness analysis in studying nanoscale drug delivery systems.

## Supporting Information Available

Information on materials, formulation procedure, experimental details on DLS, ELS, SEM, sample preparation, HPLC measurements and method development, calculation of LC and EE as well as supporting Figures S1–S5 and supporting Tables S1–S6 containing experimental and evaluated data (PDF)
