# Supplementary material for: Nanoparticle Formulation Composition Analysis by Liquid Chromatography on Reversed-Phase Monolithic Silica
Source: Anal Chem. 2022 Dec 22;95(2):565–9. doi: 10.1021/acs.analchem.2c04277 (PMC9850345; doi:10.1021/acs.analchem.2c04277)
Supplement: Supplementary file 1 — ac2c04277_si_001.pdf [file ac2c04277_si_001.pdf]

Supporting Information

Nanoparticle Formulation Composition Analysis by Liquid Chromatography on Reversed-Phase Monolithic Silica

Ekaterina Tsarenko,[a,b] Ulrich S. Schubert,[*,a,b] and Ivo Nischang[*,a,b]

[a]Laboratory of Organic and Macromolecular Chemistry (IOMC), Friedrich Schiller University Jena, Humboldtstr. 10, 07743 Jena, Germany

[b]Jena Center for Soft Matter, Friedrich Schiller University Jena, Philosophenweg 7, 07743 Jena, Germany

*Corresponding Author

Ulrich S. Schubert – Laboratory of Organic and Macromolecular Chemistry (IOMC) and Jena Center for Soft Matter (JCSM), Friedrich Schiller University Jena, 07743 Jena, Germany; orcid.org/0000-0003-4978-4670; *Email: ulrich.schubert@uni-jena.de

Ivo Nischang – Laboratory of Organic and Macromolecular Chemistry (IOMC) and Jena Center for Soft Matter (JCSM), Friedrich Schiller University Jena, 07743 Jena, Germany; orcid.org/0000-0001-6182-5215; Phone: +49-3641-948-569; *Email: ivo.nischang@uni-jena.de

Table of content

1. Detailed experimental procedures....S2
   1.1. Materials....S2
   1.2. Nanoparticle (NP) formulation....S2
   1.3. Dynamic light scattering (DLS) and electrophoretic light scattering (ELS)....S2
   1.4. Scanning electron microscopy (SEM)....S3
   1.5. Sample preparation for HPLC analysis....S3
   1.6. High performance liquid chromatography (HPLC)....S3
   1.7. HPLC method development....S4
   1.8. Loading capacity (LC) and encapsulation efficiency (EE)....S4
2. Supporting Figures S1 – S5....S5
3. Supporting Tables S1 – S6....S8
4. References....S10

1. Detailed experimental procedures

1.1. Materials

Acid-terminated PLGA (Resomer® RG 502 H, comonomer ratio 50:50, molar mass 7-17 kg mol^{-1}) for NP formulation was purchased from Evonik (Essen, Germany). Partly hydrolyzed poly(vinyl alcohol) (PVA) Mowiol 4-88, dexamethasone (Dex), dexamethasone 21-acetate (DexAce) as well as the solvents dimethyl sulfoxide (DMSO) and acetone were all purchased from Sigma Aldrich (Steinheim, Germany). Ibuprofen (Ibu) was purchased from TCI (Tokyo, Japan). HPLC grade acetonitrile (ACN) and water were obtained from VWR (Darmstadt, Germany). Ultrapure water for NP formulation experiments was acquired from a Thermo Scientific Barnsted™ GenPure™ xCAD Plus water purification system (Thermo Electron LED GmbH, Langenselbold, Germany).

1.2. Nanoparticle (NP) formulation

NPs were prepared using a previously reported nanoprecipitation formulation method.[1] Therefore, 50 mg of polymer, i.e., PLGA, was dissolved in 5 mL of acetone. Solutions of Ibu, Dex, and DexAce were prepared at a concentration of 5.0 mg mL^{-1} in acetone. Subsequently, 300 µL of the respective drug solution was mixed with the polymer solution. As the aqueous phase, 25 mL of a 0.3% (w/w) PVA solution was transferred to the organic solution (5.3 mL) containing drug and PLGA while stirring at 800 rpm at room temperature. The mixtures were left stirring overnight to evaporate acetone. Afterward, the NPs were purified by centrifugation using a 5804 R centrifuge (Eppendorf, Hamburg, Germany) spun at 10,000 rpm for 60 min at a temperature of 20 °C. The supernatant was discarded and each NP sample was resuspended with 5 mL water. Therefore, the solutions were sonicated for 15 min and vortexed for around 15 s. The solutions were stored overnight in the fridge at a temperature of 5 °C. For determination of the formulation concentration as well as for further HPLC measurements, aliquots of 400 µL of NP batches were freeze-dried (Lyophilizer Christ Alpha 2–4 LD plus, Osterode, Germany). The mass of the formulations was determined by using a microbalance (MYA 11.4Y, Radwag Waagen, Radom, Poland).

1.3. Dynamic light scattering (DLS) and electrophoretic light scattering (ELS)

The size, polydispersity index (PDI), and zeta-potential of purified and resuspended NPs were measured using a Zetasizer Nano ZS utilizing a laser with a wavelength of λ = 633 nm (Malvern Panalytical GmbH, Kassel, Germany). Intensity fluctuations were recorded at a backscattering angle of 173°. For DLS measurements an aliquot of 10 µL of the NP batch was diluted with 90 µL pure water in polystyrene UV cuvettes (Brand GmbH + Co KG, Wertheim, Germany). Each measurement was performed with the following settings: measurement temperature 25 °C, three runs of each sample with 30 s of equilibration time and 30 s of acquisition time. The intensity-weighted hydrodynamic size (z-average) distributions are shown in respective figures. The z-average hydrodynamic size, $d_{h,z}$, is accompanied with a polydispersity index (PDI) of the NPs. For zeta-potential measurements, polycarbonate cuvettes DTS1070 with gold-plated copper electrodes (Malvern Instruments Ltd., Malvern, Worcestershire, UK) were used and an 80 µL NP aliquot was diluted with 720 µL of water.

1.4. Scanning electron microscopy (SEM)

For SEM, a sample aliquot of 10 µL was spotted on a silicone substrate. Afterward, the samples were coated with a 4 nm layer of platinum via sputter coating (CCU-010 HV, Safematic, Zizers, Switzerland). Imaging of NPs was performed with a Sigma VP Field Emission SEM (Carl-Zeiss, Jena, Germany) using an InLens detector with an acceleration voltage of 6 kV. For the processing of SEM images, the software ImageJ was used.

1.5. Sample preparation for HPLC analysis

The lyophilized NP samples were dissolved in 50 µL DMSO. For this purpose, the solutions were sonicated for 3 min at room temperature. Afterward, 855 µL ACN was slowly added to the solution. The solution was mixed with the micropipette and sonicated for 1 min. In the end, 95 µL water was added and the sonication was repeated. A new solvent was prepared by mixing DMSO, ACN, and water at the same ratio, i.e., 5/85.5/9.5 (%, v/v/v) and was used for the dilution of every stock solution to the required concentration for the analysis.

For analytical recovery experiments, four lyophilized samples of the freshly formulated Ibu-loaded NPs were dissolved according to the procedure described above. Those were diluted up to the concentration of 3.0 mg mL^{-1} in DMSO, ACN, and water (5/85.5/9.5 (%, v/v/v)), followed by combination to one sample stock solution of larger volume. The spiking stock solution contained 100 µg mL^{-1} Ibu and 8.0 mg mL^{-1} PLGA. Preparation of the spiked samples was performed by adding of precise volumes of spiking stock solution and solvent, in order to achieve desired concentrations for spiking: 2.5, 5.0, and 10.0 µg mL^{-1} of Ibu and 0.2, 0.4, and 0.8 mg mL^{-1} of PLGA.

In experiments for free drug analysis, the lyophilized and stored samples of NPs were resuspended in water to reach a concentration of 3.0 mg mL^{-1}. 700 µL of this suspension was centrifuged for 1 hour at 10.000 rpm in order to sediment the particles. The supernatant was carefully collected, freeze-dried, and placed in a vacuum oven for 48 hours to evaporate the solvent. Afterward, as for NP analysis (vide supra), 100 µL of the DMSO/ACN/water (5/85.5/9.5, %, v/v/v) mixture was added to the sample followed by 1 min sonication in accordance to the previous sample preparation procedure.

1.6. High performance liquid chromatography (HPLC)

For the dissolved NP composition analysis an UltiMate 3000 Dionex UHPLC chromatographic system from Thermo Fisher Scientific (Waltham, MA, USA), equipped with a binary pump, an autosampler with temperature control, and a column oven was used. Elution was monitored via a diode array detector (DAD) at two different wavelengths, i.e., 225 nm and 254 nm, simultaneously. The autosampler temperature was set to 19 °C and the column oven temperature was set to 40 °C. All experiments comprised a Chromolith® High Resolution RP-18 endcapped monolithic column from Merck KGaA (Darmstadt, Germany) as the stationary phase. The column had a nominal length of 100 mm at an internal diameter of 4.6 mm. Those columns feature micrometer-sized flow through pores of approx. 1.1 µm, confined by a continuous mesoporous C18-derivatized skeleton containing approx. 15 nm sized mesopores. The injection volume was 10 µL and a flow rate of 1 mL min^{-1} was utilized. Mixtures of ACN/water (%, v/v) were used as the mobile phase for sample elution. For the chromatographic experiments, the lyophilized samples of NPs were dissolved according to the detailed procedure as described in Section 1.5. Before chromatographic analysis, all samples were filtered over a 0.45 µm pore size PTFE-Filter (13 mm SimplePure filter, AppliChrom Gmb, Oranienburg, Germany). In model experiments, the filtration step was seen to not affect the analytical result by only a small loss of Ibu and PLGA

material of less than 1% when comparing filtered versus unfiltered samples. Each measurement was repeated three times to calculate the standard deviation. The Thermo Scientific™ Dionex™ Chromeleon™ 7 Chromatography Data System software was used for data evaluation.

1.7. HPLC method development

For HPLC analysis of multicomponent NP systems containing drug and polymer, gradient elution programming was chosen as a starting point as all the components of the system differ in hydrophobicity, structure, and molar mass. The separation method was developed on the model system, i.e., Ibu-loaded PLGA NPs and unloaded NPs as the control. For method development an Agilent Technologies 1200 series chromatographic system from PSS (Polymer Standards Service GmbH, Mainz, Germany) with a UV absorbance detector was used. The column was placed in a TCC 6000 column oven from PSS (Polymer Standards Service GmbH, Mainz, Germany) tempered to 40 °C. The overview of all the tested, respectively, used elution programming methods according to Figure S3 is the following:

Slow increase of the ACN content in the mobile phase from 20 to 100% (v/v) leads to elution of both components from the column within 12 minutes. Here, the PLGA elutes as a broad, fronting peak at 100 % ACN in the mobile phase with some more hydrophilic (or lower molar mass) components of PLGA eluting faster than the major population and interfering with the eluting drug component (Figure S3A). Increasing gradient steepness resulted in co-elution of the drug and the more hydrophilic (lower molar mass) PLGA polymer subfractions in the front (Figure S3B). To resolve this issue, we designed a step gradient elution program, where an initial gradient with an isocratic hold at 60/40 ACN/water (%, v/v) was followed by a second gradient toward 100 % ACN in the mobile phase (Figure S3C). Since gradient elution is not really necessary for small drug molecules and the PLGA elutes at ACN contents larger 60 % (v/v) in the mobile phase, we opted for an isocratic hold at 60 % (v/v) in the mobile phase until elution of the small drug molecules under isocratic conditions with high efficiency, immediately followed by a sharp increase of the ACN content to 100%. This also results in the elution of the PLGA as a compressed, though fronting peak, with a greatly enhanced signal-to-noise ratio desired for quantification of its total amount (Figure S3D).

Thus, the final optimized method comprised an isocratic hold for 3 min at 60/40 ACN/water (%, v/v) in which the drug components eluted, followed by a steep gradient toward 100% ACN within 0.25 min to elute the PLGA. The ACN content was hold constant at 100% for 3.25 min. After that, reconditioning of the column toward injection conditions was pursued by decreasing the ACN content in the mobile phase to injection conditions within 3.50 min. After a constant hold for at least 5 min, the next injection was pursued. Elution of all components was completed within 6 min, while the total run time including conditioning for the next run was 15 min.

1.8. Loading capacity (LC) and encapsulation efficiency (EE)

LC and EE for each NP batch were calculated according to the equations below:[2,3]

$$LC = \frac{mass\ of\ drug\ recovered}{mass\ of\ polymer\ recovered} \times 100\% \qquad (S1)$$

$$EE = \frac{LC}{LC\ theoretical} \times 100\% \qquad (S2)$$

2. Supporting Figures S1 – S5

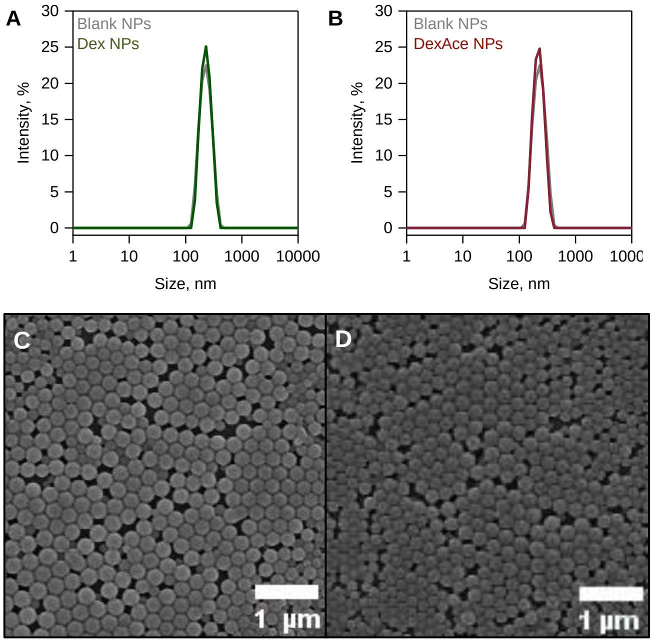

Figure S1. Intensity-based hydrodynamic size of blank PLGA NPs and NPs loaded with (A) dexamethasone (Dex) and (B) dexamethasone acetate (DexAce), respectively. SEM images of the (C) Dex- and (D) DexAce-loaded PLGA NPs, respectively.

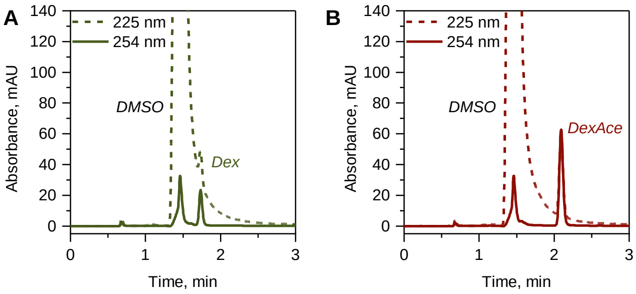

Figure S2. Establishing suitable wavelengths for (A) Dex and (B) DexAce detection in the dissolved NP samples. DAD operated at 254 nm provides baseline separation of the drug and DMSO peaks while the lower wavelength of 225 nm shows strong DMSO absorption. Measurement conditions: flow rate 1 mL min^{-1}, isocratic hold of 3 minutes at 60% (v/v) of ACN in the mobile phase, after which a linear gradient of ACN (from 60% (v/v) to 100% in 0.25 min was programmed.

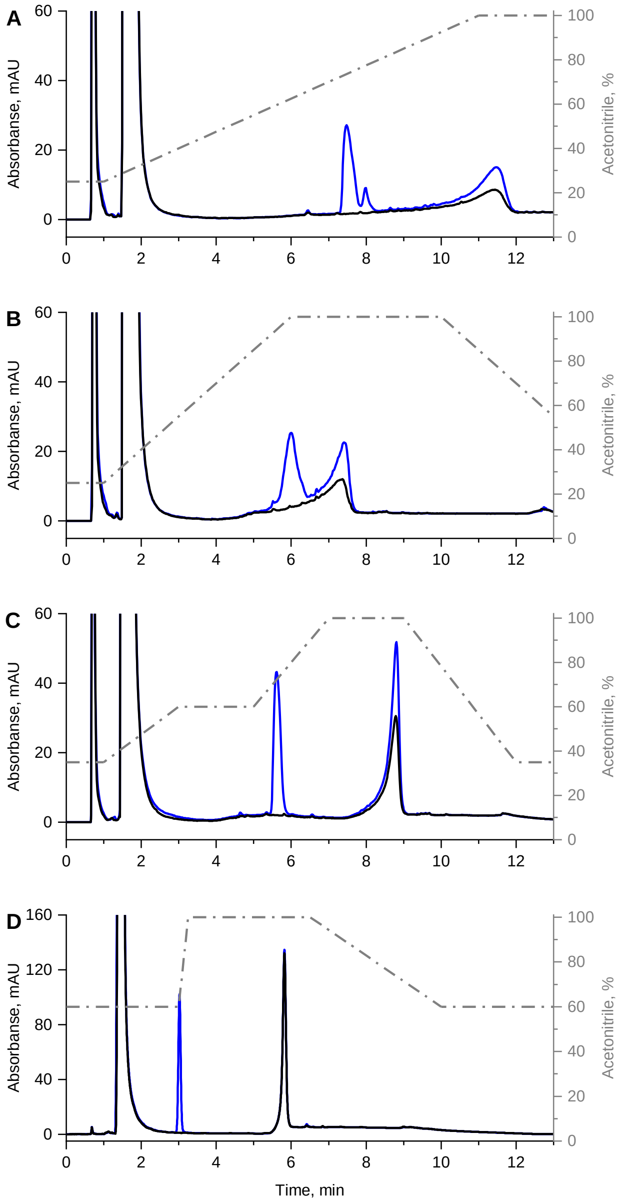

Figure S3. Development of the compositional analysis method for the model drug delivery system PLGA NPs with Ibu (blue) and without Ibu (black) with gradient programming shown by the grey dot-dashed lines. (A) Simple linear gradient in 10 min. (B) Simple linear gradient in 5 min. (C) First gradient after an isocratic hold, followed by a second gradient after an isocratic hold. (D) Isocratic hold for 3 min allowing for drug elution followed by a steep gradient. Measurement conditions: flow rate 1 mL min^{-1}, UV detection at 225 nm.

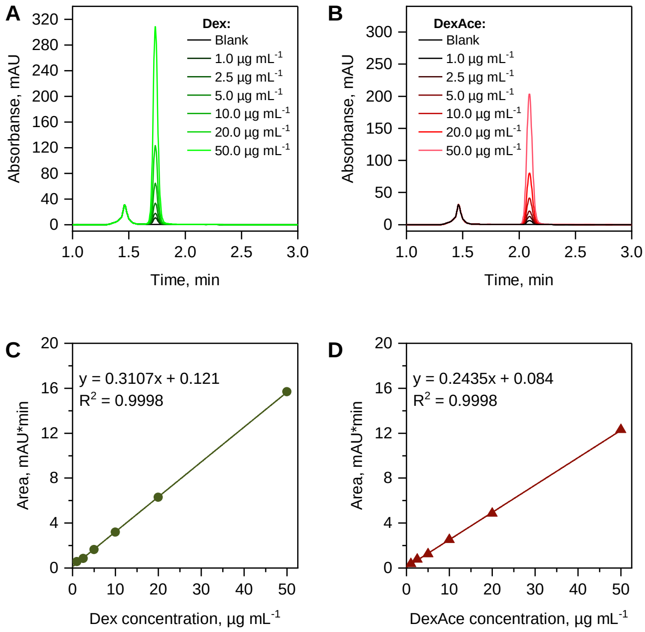

Figure S4. Elugram of (A) Dex (1.0 – 50 µg mL^{-1}) and (B) DexAce (1.0 – 50 µg mL^{-1}) standards detected via DAD at 254 nm. Calibration curves for (C) Dex and (D) DexAce presented by plotting peak areas as the function of analyte concentrations. Same elution conditions as in Figures 2 and S2.

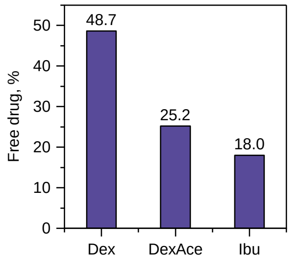

Figure S5. Results from resuspension experiments of lyophilized samples followed by centrifugation and supernatant analysis with the exactly same sample lyophilization, dissolution, and preparation procedure (see Section 1.5 of the Supporting Information). Same elution conditions as in Figures 2 and S2.

3. Supporting Tables S1 – S6

Table S1. Hydrodynamic size, $d_{h,z}$, and its variation, the polydispersity index (PDI), and the ζ-potential for each NP batch measured by DLS and ELS.

Formulation	$d_{h,z} \pm \Delta d_{h,z}$, nm	PDI	ζ-potential, mV
Blank NPs	221±34	0.023	-28.17
Dex NPs	225±17	0.006	-30.19
DexAce NPs	221±23	0.011	-31.53
Ibu NPs	230±37	0.026	-23.24

Table S2. Chromatographic figures of merit of analyzed drug components measured during an isocratic hold at 60/40 ACN/water (%, v/v)*. Elution conditions: flow rate 1 mL min^{-1}, UV absorption detection at 225 nm (Ibu) and 254 nm (Dex and DexAce). Same elution conditions as in Figures 2 and S2.

Component	t_R[a], min	Asymmetry	Efficiency, plates/m	HETP[b], µm
Dex	1.73	1.16	81000	12.3
DexAce	2.09	1.12	81000	12.4
Ibu	3.02	1.04	108000	9.3

[a] retention time, [b] height equivalent to a theoretical plate

* 3 min of isocratic hold followed by linear gradient of ACN (from 60% (v/v) to 100%) in 0.25 min

Table S3. Compositional analysis of formulated NPs. The values were calculated using the calibration data (Figure 3B and C and Figure S4C and D) with the standard deviation of three repetitive injections (in brackets) for each sample. Same elution conditions as in Figures 2 and S2.

NP sample	Drug, µg mL^{-1}	PLGA, mg mL^{-1}
Dex NPs	3.100 (± 0.003)	2.762 (± 0.001)
DexAce NPs	14.419 (± 0.001)	2.712 (± 0.001)
Ibu NPs	21.090 (± 0.020)	2.698 (± 0.002)

Table S4. Determination of amount, repeatability, and recovery of Ibu and PLGA in solutions of Ibu-loaded NP formulations with the standard deviation of three repetitive injections (in brackets). Same elution conditions as in Figures 2 and S2.

Component	Content	Spiked amount	Amount found	Recovery, %
PLGA		0.200	2.729 (± 0.009)	101.6
[mg mL^{-1}]	2.526 (± 0.003)	0.400	2.941 (± 0.013)	103.8
		0.800	3.327 (± 0.013)	100.1
Ibu		2.500	15.986 (± 0.101)	93.9
[μg mL^{-1}]	13.639 (± 0.059)	5.000	18.636 (± 0.063)	99.9
		10.000	23.377 (± 0.043)	97.4

Table S5. Sample preparation for recovery experiments.

Spiked amount		Sample[a], μL	Spiking solution[b], μL	Solvent[c], μL
Ibu, μg mL^{-1}	PLGA, mg mL^{-1}			
0	0	900	0	100
2.5	0.2	900	25	75
5.0	0.4	900	50	50
10.0	0.8	900	100	0

[a] 3.0 mg mL^{-1} solution of dissolved Ibu-loaded NPs; [b] contains 100 μg mL^{-1} Ibu and 8.0 mg mL^{-1} PLGA;
[c] DMSO/ACN/water 5/85.5/9.5 (%, v/v/v)

Table S6. Composition analysis of filtered and unfiltered model samples containing Ibu and PLGA mixture dissolved in DMSO/ACN/water 5/85.5/9.5 (%, v/v/v). Same elution conditions as in Figures 2 and S2.

Component	Non-filtered sample	Filtered sample	Difference
Ibu [μg mL^{-1}]	25.59 (± 0.03)	25.39 (± 0.03)	0.20
PLGA [mg mL^{-1}]	2.040 (± 0.002)	2.021(± 0.002)	0.019

4. References

(1) Martinez Rivas, C. J.; Tarhini, M.; Badri, W.; Miladi, K.; Greige-Gerges, H.; Nazari, Q. A.; Galindo Rodriguez, S. A.; Roman, R. A.; Fessi, H.; Elaissari, A. *Int. J. Pharm.* **2017**, *532*, 66-81.
(2) Behnke, M.; Vollrath, A.; Klepsch, L.; Beringer-Siemers, B.; Stumpf, S.; A. Czaplewska, J.; Hoeppener, S.; Werz, O.; Schubert, U. S. *Polymers* **2020**, *12*, 2751.
(3) Shkodra-Pula, B.; Kretzer, C.; Jordan, P. M.; Klemm, P.; Koeberle, A.; Pretzel, D.; Banoglu, E.; Lorkowski, S.; Wallert, M.; Höppener, S.; et al. *J. Nanobiotechnology* **2020**, *18*, 73.
